# Microbiota and horizontal microbial transmission dynamics associated with bramble (*Rubus* spp.) flowers

**DOI:** 10.1186/s40793-026-00853-3

**Published:** 2026-01-17

**Authors:** Haoran Shi, Stefan Ratering, Bellinda Schneider, Dirk Höper, Sylvia Schnell

**Affiliations:** 1 Institute of Applied Microbiology, Justus-Liebig University Giessen, IFZ Heinrich-Buff Ring 26, 35392 Giessen, Germany; 2 Friedrich-Loeffler-Institut, Federal Research Institute for Animal Health, Südufer 10, 17493 Greifswald, Insel Riems Germany

**Keywords:** Floral microbiota, Horizontal microbial transmission, Bramble, Hub plant, Host-microbe interaction

## Abstract

Microbes are integral players in plant–insect networks, and play crucial roles in host health and ecological interactions. However, the horizontal microbial transmission dynamics via flowers remain insufficiently understood, particularly in hub plants visited by diverse insect communities. Here, using 16S rRNA gene and ITS2 metabarcoding, we characterized the bacterial and fungal communities associated with open flowers, insect-excluded bagged flowers, and flower buds of bramble (*Rubus* spp.), a highly insect-visited hub plant. Our results show that environmental deposition and insect visitation significantly altered floral microbiota diversity and microbial load, particularly for bacterial communities. Insect visitation potentially enriched fermentative and probably also pathogenic bacteria, including *Spiroplasma*. While bagged flowers and open flowers both showed high within-group variation for bacterial communities, the microbial networks were more internally connected in bagged flowers, and more centralized in open flowers. The distinct network properties might be due to flower group-specific microbial hub genera introduced by different horizontal transmission routes. Moreover, cross-domain network analysis revealed hub genera unique to bacteria-fungi interactions, including *Cladosporium*, which was consistently detected across all flower groups. These findings highlight the dominant role of insect visitation in shaping floral microbiota and underscore the ecological significance of hub plants in horizontal microbial transmission across plant–pollinator networks.

**Supplementary Information:**

The online version contains supplementary material available at 10.1186/s40793-026-00853-3.

## Background

Microbes are functionally essential players for both plant and insect health. In plants, associated microbes can promote growth, facilitate nutrient uptake, and confer tolerance and resistance to environmental stressors and pathogens, and these microbe-mediated benefits are also observed in insects [1, 2]. As plants ubiquitously interact with insects in diverse ecological systems, microbes are inevitably transmitted between them, and one of the microbial transmission routes is via pollination. The microbes transmitted to flowers depend on the microbial pool carried by visiting insects [3, 4], as well as the assemblage of visiting insects filtered by various flower cues such as volatile organic compounds, floral morphology, floral color, and nutritional content [5–8]. The physical and chemical properties of plant flowers can further influence the survival and establishment of epiphytic microbes [6, 9, 10].

Pathogenic microbes can exploit plant–insect networks for their dispersal. For example, several bee-associated pathogens, including microsporidian fungi *Vairimorpha* species (*V. apis* and *V. cerane*), mycoplasma-like bacteria *Spiroplasma* species (*S. apis* and *S. melliferum*), and ascomycetous fungus *Ascosphaera*, have been shown to utilize the floral transmission routes [11–14]. These pathogens can cause various diseases and pose threats to both insect individuals and entire communities [15–18]. This mode of pathogen transmission can occur not only within a single insect species but also between different insect species [19]. Plant–insect networks can also aid the spread of plant-associated pathogens. *Erwinia amylovora* is a bacterial phytopathogen that causes the destructive disease fire blight in some commercially important pome fruit trees such as apple and pear [20]. This pathogen is commonly spread by honeybees from diseased to healthy flowers [21]. In addition, plant fungal pathogens such as *Microbotryum violaceum* and *Monilinia vaccinii-corymbosi* can also be vectored by visiting insects [22, 23].

Insect-pollinated plants are not equally visited by the available insects within the local pollinator community, resulting in distinct pollinator profiles. Generalist plant species with easily accessible floral morphologies and rich nutritional reward tend to attract a broad array of pollinators [24, 25]. In contrast, other plants attract only specialized insects due to co-evolved physical or chemical traits [26, 27]. Plants that host diverse pollinators are considered hubs within plant–insect networks. These highly visited plants can also serve as key nodes for horizontal microbial transmission, contributing significantly to floral-microbe associations [28, 29]. Their co-occurrences with numerous microbes additionally render them potential hotspots for the transmission of pathogens [14]. Therefore, characterizing microbial composition and horizontal transmission dynamics associated with hub plants holds significance for ecological epidemiology.

Bramble (*Rubus* spp.) belongs to the family Rosaceae and comprises hundreds of species and hybrids [30]. Economically important bramble crops include raspberry and blackberry, both of which have substantial global production [31, 32]. Bramble fruits are highly nutritious, rich in proteins and sugars, and exhibit strong antioxidant capacity, thus being considered as “superfoods” for human consumption [33, 34]. In addition, bramble is a valuable foraging resource, visited by insects from various orders including Diptera, Lepidoptera, Hymenoptera, Coleoptera [35]. A recent study analyzing plant and microbial composition in honeybee-collected pollen also identified bramble as a hub plant in plant–microbe networks and suggested its potential role in horizontal pathogen transmission [14]. Previous research on bramble microbiota has mainly focused on seeds, leaves, roots, and rhizosphere soil [36–38]. However, the floral microbiota and horizontal microbial transmission dynamics associated with bramble remain poorly understood.

In this study, we aimed to characterize the bacterial and fungal microbiota of bramble flowers using bacterial 16S rRNA gene and fungal ITS2 metabarcoding. By comparing open flowers, bagged flowers, and flower buds, we investigated the horizontal microbial transmission dynamics via this highly insect-visited hub plant. We examined microbial loads and diversity, functional traits, and microbial co-occurrence networks to better understand the underlying transmission processes.

## Methods

### Sample collection and DNA extraction

Bramble (*Rubus* spp.) flowers were collected in three locations (50.57 N 8.67 E; 50.62 N 8.64 E; 50.75 N 8.49 E) around the Giessen area, Hesse, Germany, in July 2024 around the same time, and allocated into three flower groups: open flowers, bagged flowers, and flower buds. The microbiota composition of these flower groups is primarily influenced by insect visitation, airborne deposition, and the indigenous microbiota of bramble, respectively. No systematic surveys of the insect community visiting open flowers were conducted during this study. Open flowers and flower buds were directly placed into 50-ml sterile tubes upon collection. For the collection of bagged flowers, flower buds were enclosed in mesh bags (pore size approximately 1 mm × 1 mm; Organzabeutel24, Germany) to exclude insect visitation for up to two weeks and were collected at full bloom. At each location, three to four flowers (open or closed) were sampled from different branches of the bramble thicket and later pooled. In total, 27 samples (9 samples per flower group) were collected and stored at − 20 °C until further processing. For DNA extraction, samples were frozen in liquid nitrogen and mechanically homogenized into fine powder. Metagenomic DNA extraction was then performed using the NucleoSpin Plant II kit (Macherey–Nagel GmbH, Germany) according to the manufacturer’s instruction with a prior zirconia bead-beating step using FastPrep-24 tissue and cell homogenizer (MP Biomedicals, USA) at 5.5 m s^−1^ for 45 s.

### Quantitative PCR

Total bacterial and fungal loads in the floral metagenomic DNA were quantified by quantitative real-time PCR. For bacterial quantification, primers 520F (5′-AYTGGGYDTAAAGNG-3′) [39] and 907R (5′-CCGTCAATTCMTTTRAGTTT-3′) [40] targeting the 16S rRNA gene were used, along with peptide nucleic acids (PNAs) to block the amplification of mitochondrial (5′-AAACCAATTCACTTGAGT-3′) [41] and plastid sequences (5′-GGCTCAACCCTGGACAG-3′) [42]. For fungal quantification, primers ITS5 (5′-GGAAGTAAAAGTCGTAACAAGG-3′) and ITS2 ngs (5′-TTYRCKRCGTTCTTCATCG-3′) [43] targeting the ITS1 region were used in combination with a blocking primer containing a 3′ C3 spacer to block the amplification of *Rubus* ITS1 (5′-GATCATTGTCGAAACCTGCCCAGCAG-3′) [36]. To normalize bacterial and fungal loads, the plant transcription elongation factor (*tef*) gene was quantified using primers tef_f (5′-ACTGTGCAGTAGTACTTGGTG-3′) and tef_r (5′-AAGCTAGGAGGTATTGACAAG-3′) [44].

The volume of each reaction was 10 µl, consisting of 1 × ABsolute qPCR SYBR Green Mix (Thermo Scientific, USA), 300 nM of each primer, 1 μM of each PNA or 10 μM of blocking primer if required, and 1 μl DNA template. Amplification was performed using a Rotor Gene Q (QIAGEN GmbH, Germany) with program settings described in Table S1. Following amplification, a melting curve analysis was performed by gradually increasing the temperature from 60 to 95 °C with fluorescence measurements taken at 1 °C intervals to confirm the specificity of the qPCR products. The copy numbers of standard DNA samples were calculated according to [45], and the calibration curves were constructed using tenfold serial dilutions of DNA standards ranging from 10^1^ to 10^6^ copies in four replicates. The gene copy numbers in real samples were obtained in Q-Rex software (version 1.1.04). Bacterial and fungal loads were calculated as the ratio of microbial gene copy numbers to *tef* gene copy numbers, and log2-transformed for statistical analysis and visualization. The differences in microbial loads across the flower groups were assessed using the Wilcoxon test with Bonferroni correction.

### Bacterial and fungal metabarcoding

The 16S rRNA gene V4-V5 regions and the ITS2 region were used as barcodes to profile the bacterial and fungal microbiota, respectively. The 16S rRNA gene was amplified using the same primers as in the quantitative PCR. The ITS2 region was amplified using primers ITS3 KYO2 forward (5′-GATGAAGAACGYAGYRAA-3′) [46] and ITS4 reverse (5′-TCCTCCGCTTATTGATATGC-3′) [43]. Amplification, purification, and library preparation for the 16S rRNA gene followed the protocols described by [14], while the procedures for the ITS2 followed [47]. A negative control consisting of PCR-grade water processed through all DNA extraction, amplification, and library preparation steps was included to confirm the absence of contamination. Prepared libraries were sequenced using Ion Torrent S5 (Thermo Fisher Scientific, Waltham, MA, USA), and the sequencing data were deposited in the NCBI Sequence Read Archive under the BioProject PRJNA1261409 and PRJNA1261414.

We used QIIME 2 amplicon distribution (released in 2024.10) for sequencing data processing. Demultiplexed sequences were imported into QIIME 2, and the ITS2 sequences were trimmed using the ITSxpress plugin [48]. The dada2 plugin was then used to denoise and dereplicate the sequences into amplicon sequence variants (ASVs) [49]. The 16S rRNA gene sequences were truncated at the 300th position, while the ITS2 sequences were not truncated due to their highly variable lengths [50]. Taxonomy assignment of ASVs was performed using sklearn-based classifiers [51, 52] pre-trained with SILVA database (version 138.1) [53] for the 16S rRNA gene sequences and EUKARYOME database (version 1.9.4) [54] for the ITS2 sequences. ASVs identified with non-bacterial or non-fungal origins were excluded from downstream analyses.

### Bioinformatic analyses

The taxonomy information was refined by replacing the unknown or uninformative taxonomy with the first informative taxonomy from a higher taxonomic rank, and taxonomy barplot at genus level was generated using the R package microViz (version 0.12.6) [55]. The shared and unique genera were analyzed using the R package UpSetR (version 1.4.0) [56]. Functional annotation of bacterial and fungal genera was performed using FAPROTAX database (version 1.2.10) and FungalTraits database (version 1.2), respectively [57, 58]. Functional traits of bacteria were grouped into functional guilds chemotroph, phototroph, pathotroph, biogeochemical cycle (including guilds related to carbon, nitrogen, sulfur, iron, and manganese cycling), and fermentation (including both obligate and facultative fermenters), and functional traits of fungi were grouped into saprotroph (decomposing dead cells), pathotroph (obtaining nutrition from living host cells and cause disease), and symbiotroph (exchanging resources with host cells) [59, 60]. Taxa with multiple functions were assigned to all applicable categories. Alpha diversity analysis at genus level was performed with the richness index using the R package MeanRarity (version 0.0.1.4) [61]. Beta diversity analysis at genus level was performed based on robust Aitchison distances and visualized through principal component analysis (PCA) ordination [62]. Differences across the flower groups were assessed by permutational multivariate analysis of variance (PERMANOVA) [63] and permutation test for homogeneity of multivariate dispersions (PERMDISP) [64] with 999 permutations using the R packages microViz and vegan (version 2.6–4) [65]. Pairwise differences were assessed using the Wilcoxon test with Bonferroni correction.

Differentially abundant bacterial and fungal genera were identified using the R package ANCOMBC (version 2.0.3) [66]. Benjamini–Hochberg correction was used to adjust *p*-values and control the mixed directional false discover rate. Pseudo-count sensitivity analysis was performed as recommended by the package developer. Genera with *p*-values lower than 0.05 and unaffected by pseudo-count choice were designated as differentially abundant genera. As ANCOMBC excluded low-prevalence genera, Fisher’s exact test based on presence/absence of microbial genera with Benjamini–Hochberg correction was performed for compensation.

Bacterial, fungal and cross-domain co-occurrence networks at genus level were constructed using the web-based application SpeSpeNet [67]. In detail, the issues of compositionality and sparsity in amplicon sequencing data were mitigated using SparCC algorithms [68] and imputation of random pseudo-counts [69]. Occurrence and abundance thresholds were set at 2 and 0.1%, respectively. Only correlations with edge weights above 0.2 were included in network construction. Resulting networks were imported into Cytoscape (version 3.9.1) for the analysis of network attributes, including number of nodes and edges, average number of neighbors, characteristic path length, network density, clustering coefficient, and network centralization [70]. In each network, genera within the top 10% for both degree and betweenness centrality were assigned as hub genera.

## Results

### Bacterial and fungal composition of bramble flowers

**Fig. 1:**
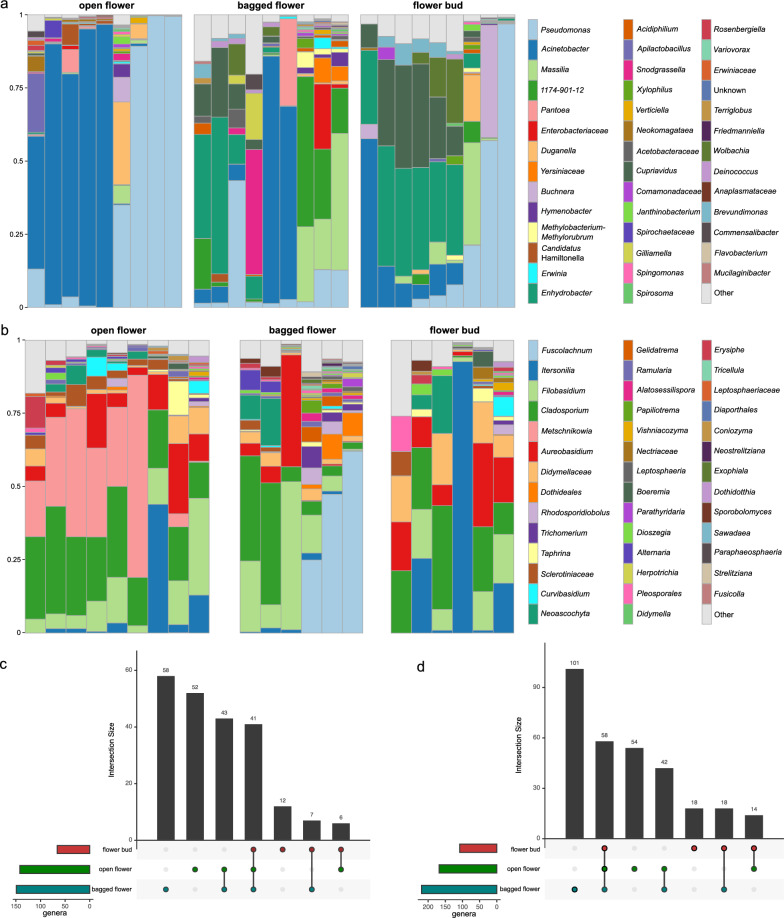
Bacterial and fungal composition in open flowers, bagged flowers, and flower buds of bramble. **a**, **b** Taxonomy barplots showing the relative abundance of bacteria (**a**) and fungi (**b**) across different flower groups. ASVs were collapsed to genus level and a higher taxonomic rank was used when genus was unavailable. Genera with relative abundance lower than 0.5% were grouped into “Other”. **c**, **d** UpSetR graphs showing the unique and shared bacterial (**c**) and fungal (**d**) genera across different flower groups

To determine the influence of horizontal transmission including environmental deposition and insect visitation on the composition of bramble floral microbiota, bacterial and fungal communities across flower buds, open flowers (insect-visited), and bagged flowers (insect-excluded) were compared. As flower buds are relative isolated from the environment, it was hypothesized that open flowers and bagged flowers harbored more microbial genera compared to flower buds. After sequence quality control and removal of non-target sequences, the bacterial dataset contained 336,673 sequences (mean: 12,469 sequences per sample), and the fungal dataset contained 169,628 sequences (mean: 6283 sequences per sample). Six samples were removed for fungal analysis due to low sequencing depth. Rarefaction curves plateaued for all remaining samples, indicating that sequencing depth was sufficient to capture the majority of microbial diversity (Fig. S1). The remaining sequences were grouped into 687 bacterial ASVs and 667 fungal ASVs, which were further collapsed into 219 bacterial genera and 307 fungal genera. We found that 88.5% bacterial ASVs and 87.6% fungal ASVs could be classified to genus level. However, only 44.4% bacterial ASVs and 64.9% fungal ASVs could be classified to species level. Due to the low taxonomic resolution on species level, all following analyses will be performed on genus level. The most prevalent bacterial genera (classes) included *Acinetobacter* (*Gammaproteobacteria*), *Cupriavidus* (*Betaproteobacteria*), and *Enhydrobacter* (*Gammaproteobacteria*), each identified in at least 92.6% of the samples (Fig. 1a). The most prevalent fungal genera were *Cladosporium* (*Dothideomycetes*), *Aureobasidium* (*Dothideomycetes*), and *Filobasidium* (*Tremellomycetes*), present in at least 95.2% of the samples (Fig. 1b). As expected, open flowers (142 bacterial and 168 fungal genera) and bagged flowers (149 bacterial and 219 fungal genera) harbored more bacterial and fungal genera compared to flower buds (66 bacterial and 108 fungal genera), with 41 bacterial genera and 58 fungal genera shared across all three groups. Open and bagged flowers contained a considerable number of unique bacterial and fungal genera, while flower buds harbored the fewest unique microbial genera (Fig. 1c and d).

### Microbiota structure and microbial load across bramble flower groups

**Fig. 2:**
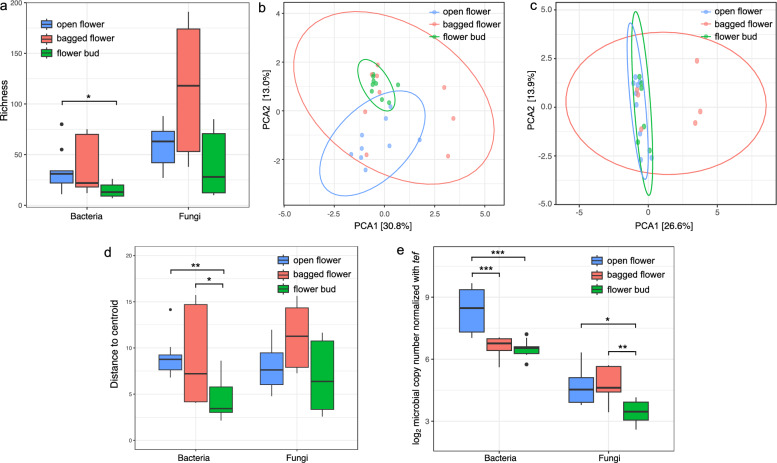
Microbial diversity and abundance in open flowers, bagged flowers, and flower buds of bramble. **a** Richness of bacterial and fungal communities at genus level across different flower groups. **b**, **c** Beta diversity analysis of bacterial (**b**) and fungal (**c**) communities at genus level across different flower groups based on robust Aitchison distances. The ellipses represent the 95% confidence intervals for each group. **d** Distances from each sample to the group centroid in the bacterial and fungal beta diversity plots. Larger distance indicates more pronounced within-group variation. **e** Microbial load of bacteria and fungi relative to plant *tef* gene quantified by quantitative real-time PCR across different flower groups. Wilcoxon test with Bonferroni correction was performed. * *p* ≤ 0.05, ** *p* ≤ 0.01, *** *p* ≤ 0.001

Following compositional characterization, the influence of horizontal microbial transmission on the bramble floral microbiota was further elucidated by assessing microbial diversity, community structure, and microbial load. It was speculated that open flowers and bagged flowers, which experienced greater exposure to environmental sources and insect visitation, would possess increased microbial diversity and exhibit distinct community structure relative to flower buds. Open flowers subjected to insect visitation were also predicted to harbor a higher microbial load compared to other groups. These hypotheses were partially supported by the data, particularly for bacterial communities. Genus-level alpha diversity analysis indicated that bacterial richness was higher in open flowers and bagged flowers than in flower buds with significant or close-to-significant *p*-values (Fig. 2a; Table S2). Beta diversity analysis based on robust Aitchison distances also showed significant differences in bacterial community across the flower groups (PERMANOVA: F = 2.02, *p*-value = 0.009) (Fig. 2b; Table S3), which was mainly driven by significantly different within-group variations (PERMDISP: F = 5.65, *p*-value = 0.015), with open flowers and bagged flowers showing greater dispersion in community structure compared to flower buds (Fig. 2d; Table S3). No significant differences were observed across three sampling sites (PERMANOVA: F = 0.65, *p*-value = 0.941) (Fig. S2). In addition, the bacterial load in open flowers was significantly higher than that in bagged flowers (*p*-value = 0.00025) and flower buds (*p*-value = 0.00086) (Fig. 2e).

While the influence of horizontal transmission on the bacterial community was apparent, the fungal community appeared less affected. For fungi, beta diversity analysis revealed significant differences among flower groups (PERMANOVA: F = 1.89, *p*-value = 0.009) but sampling sites (PERMANOVA: F = 0.64, *p*-value = 0.993), whereas differences in within-group variation (PERMDISP: F = 2.56, *p*-value = 0.109) and alpha diversity were not significant (Figs. 2a, c, d and S2; Tables S2 and S3). Moreover, fungal load was primarily influenced by environmental deposition, as it was the lowest in flower buds compared to the open flowers (*p*-value = 0.0233) and bagged flowers (*p*-value = 0.0023). Fungal load did not differ significantly between open and bagged flowers, indicating a limited impact of insect visitation (Fig. 2e).

### Functional annotation of bacterial and fungal genera

**Fig. 3:**
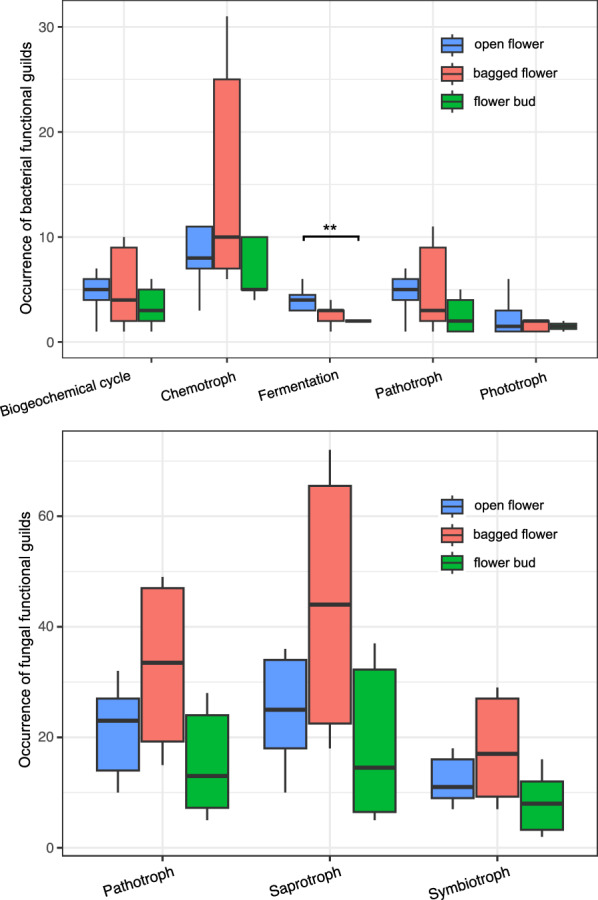
Occurrence of bacterial (upper graph) and fungal (lower graph) functional guilds in open flowers, bagged flowers, and flower buds of bramble. Functional annotation was performed at genus level using FAPROTAX database for bacteria and FungalTraits database for fungi. Wilcoxon test with Bonferroni correction was performed. ** *p* ≤ 0.01

Beyond taxonomic information, functional annotation was conducted to understand the potential ecological functions and processes associated with microbial genera across the flower groups. In total, 117 bacterial genera and 226 fungal genera were assigned at least one functional trait (Tables S4 and S5). Shared genera across the flower groups indicated their consistent associations with plants and exhibited broad functional traits, with some classified as plant pathogens such as *Ralstonia* (*Betaproteobacteria*), and *Cladosporium*. Some pathogenic microbes, such as *Spiroplasma* and members of the family *Anaplasmataceae*, were uniquely identified in open flowers, suggesting their insect-mediated transmission. Considering the compositionality of amplicon sequencing data, we compared the occurrences of different functional guilds across the flower groups instead of relative abundance (Fig. 3). The occurrence of bacterial genera with fermentation capability was significantly higher in open flowers than in flower buds (*p*-value = 0.0066), and pathogenic bacteria showed a trend toward higher occurrence in open flowers with a borderline *p*-value (*p*-value = 0.094) (Table S6). For fungal functional guilds, no significant differences were detected across the flower groups (Table S7).

### Differentially abundant genera across bramble flower groups

Surprisingly, although many unique genera were identified across the flower groups, the number of differentially abundant genera was minor (Table S8). For bacterial genera, only *Neokomagataea* (*Alphaproteobacteria*) was differentially abundant, showing enrichment in open flowers compared to both bagged flowers and flower buds. For fungal genera, *Metschnikowia* (*Saccharomycetes*) was enriched in open flowers, whereas *Cryptococcus* (*Tremellomycetes*) and *Hannaella* (*Tremellomycetes*) were differentially abundant in bagged flowers relative to the other groups. The limited amount of differentially abundant genera might be attributed to high within-group variation, which reduced the consistency of genera within each flower group. In Fisher’s exact test, no genera showed statistically significant associations with any flower group after correction for multiple testing.

### Co-occurrence network and hub genera

**Fig. 4:**
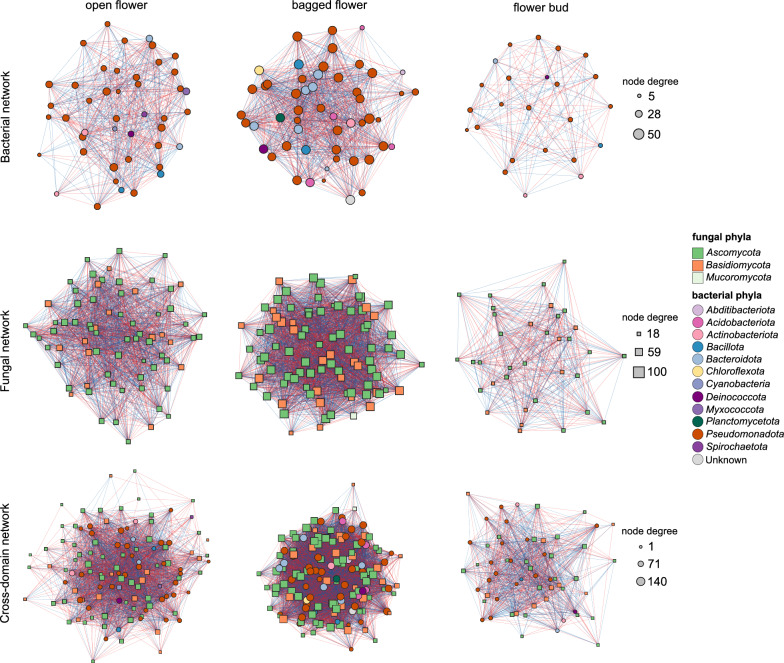
Single- and cross-domain networks from open flowers, bagged flowers, and flower buds of bramble. Each node represents a microbial genus, and node color indicates the corresponding phylum as shown in the legend. Red and blue lines indicate positive and negative co-occurrences, respectively. The node size represents node degree

Co-occurrence networks can reveal underlying microbial assembly processes and potential microbial interactions. Therefore, to assess the influence of horizontal microbial transmission via environmental deposition and insect visitation, bacterial, fungal, and cross-domain networks using the SparCC algorithm were constructed across the flower groups (Fig. 4). The networks derived from flower buds consistently exhibited the lowest number of nodes and edges, as well as the lowest average number of neighbors, indicating the networks were relatively sparse and isolated (Table S9). This is somewhat expected, as flower buds harbored notably fewer bacterial and fungal genera compared to open and bagged flowers. In addition, the bagged flower networks had the lowest characteristic path length and the highest network density and clustering coefficient, suggesting that these networks were more tightly connected and that microbes were more interactive in this environment. In contrast, the open flower networks were more centralized compared to the bagged flower networks and the flower bud networks, as reflected by higher network centralization values. The centralization of the open flower networks was particularly pronounced when cross-domain interactions were considered.

**Fig. 5:**
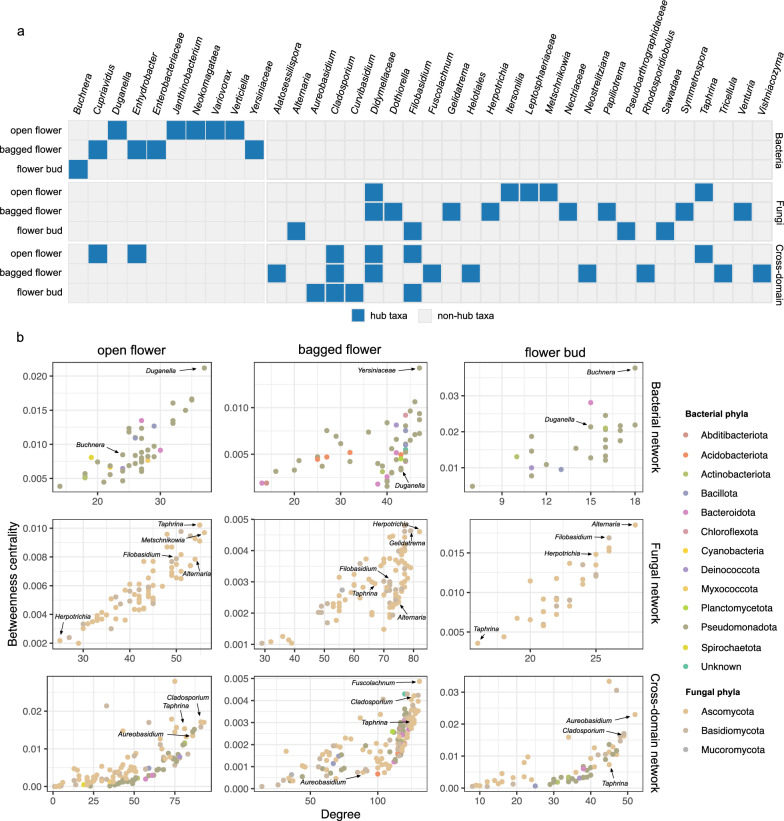
Hub genera in the single- and cross-domain networks from open flowers, bagged flowers, and flower buds of bramble. **a** Heatmap of hub genera identified in different networks based on degree and betweenness centrality. When genus-level identification is not available, the lowest available, informative higher taxonomic rank is presented. **b** Scatter plots of degree versus betweenness centrality for each node in the networks. Selected hub genera and their positions as non-hubs in other networks are indicated with arrows

Across the different networks, we identified distinct sets of bacterial and fungal hub genera, and the taxonomic identities of these hub genera were strongly influenced by the flower groups (Fig. 5a). Most hub genera were specific to a single flower group, with only a few exceptions. In the bacterial networks, five hubs were present in open flowers, four in bagged flowers and one in flower buds, with no bacterial hub genera shared across the flower groups. In the fungal networks, five hubs were detected in open flowers, eight in bagged flowers, and four in flower buds, with *Didymellaceae* (*Dothideomycetes*) as the only shared fungal hub. In the cross-domain networks, six hubs were identified in open flowers, nine in bagged flowers, and four in flower buds. *Didymellaceae* and *Filobasidium* were hubs shared between two flower groups, while *Cladosporium* was shared across all three groups. Some hub genera were uniquely present in one network but absent from the others. For example, *Metschnikowia* was a fungal hub in the open flower network, however, it was completely absent from the other fungal networks (Fig. 5b). Moreover, within each flower group, the hub genera showed distinct patterns between the single-domain networks and the cross-domain network. Many hub genera in the bacterial networks were non-hub in the cross-domain networks, and several non-hub genera in the fungal networks emerged as hubs in the cross-domain networks.

## Discussion

Horizontal microbial transmission occurs frequently in pollinator-plant networks, and hub plants that are visited by a diverse array of insects can play an important role in this process [28, 71, 72]. In this study, we characterized the floral microbiota of a highly insect-visited hub plant, bramble (*Rubus* spp.). Flower buds, bagged flowers, and open flowers were sampled to reveal the microbial communities before flowering (pre-anthesis) and how they were influenced during flowering (anthesis) by environmental deposition and insect visitation. Our results show that bramble flower buds were associated with a relatively small microbial community, which expanded and underwent marked shifts in community composition and network structure during anthesis, with insect visitation acting as a major driver of these changes.

In our bramble flower samples, flower buds were considered as the baseline, representing the pre-anthesis microbial communities.

Most microbial genera identified in flower buds were shared with open and bagged flowers, indicating that these shared microbes may adopt a lifestyle tightly associated with plants. The origins of these baseline microbes are likely diverse: certain microbes are vertically transmitted and systematically present in the plant as phytopathogens or endophytes, while some are environmental microbes passively deposited on bud surfaces via aerosols or airborne spores, particularly as surface sterilization of buds was not performed, and some microbial groups can utilize multiple transmission routes. For example, the phytopathogen *Ralstonia* can persist within plant tissues by forming biofilms and colonizing plant xylem vessels using divergent respiratory strategies [73]. Bacterial endophytes such as *Pantoea* (*Gammaproteobacteria*), *Sphingomonas* (*Alphaproteobacteria*), and *Methylorubrum* (*Alphaproteobacteria*) are consistently found in numerous plant compartments, including roots, shoots, spikes, and seeds [74], while *Sphingomonas* and *Methylorubrum* are also key members of the airborne bacterial community [75]. Some fungal plant endophytes in the baseline microbes include *Cladosporium* and *Alternaria* (*Dothideomycetes*), which have been recovered from seeds, cotyledons, leaves, and can be transmitted between plant generations via pollen [76], while these two fungal genera also include widespread airborne species [77]. In addition to plant endophytes, some insect endophytes were also identified, such as *Wolbachia* (*Alphaproteobacteria*). These insect endophytes were not removed from the dataset as contaminants, as both plant and insect endophytes demonstrate genuine ecological occurrence, and can be transmitted through plant–insect interaction. For example, insect endophyte *Wolbachia* can be deposited in plant phloem via infected whiteflies, persisting for at least 50 days, and subsequently be transmitted to uninfected whiteflies [78]. Conversely, plant endophytes community can be acquired by phloem-feeding insects such as leafhoppers and transmitted to endophyte-free plants [79]. Quantitative analyses of community composition (total and unique genera, alpha and beta diversity metrics, and microbial load) revealed that flower buds tended to have the lowest values compared to open and bagged flowers. Although the significant differences were sometimes absent in fungal communities, for example in alpha and beta diversity, this tendency is particularly evident for bacterial communities. These results suggest that limited exposure to external microbial sources restricts the microbial diversity and complexity in flower buds, resulting in a comparatively sparse and stable microbial assemblage at the pre-anthesis stage.

Upon anthesis, flowers are colonized by additional microbes through environmental deposition and insect-mediated transmission [3, 80, 81]. However, the relative contribution of different horizontal microbial transmission routes to the transmission dynamics remains insufficiently understood. Moreover, previous related studies often showed limitations, such as the lack of microbial quantification, lack of community structure information, or the use of analytical methods that do not account for the compositionality of amplicon sequencing data [80, 82, 83]. Here, we found that bacterial diversity expanded substantially in both open and bagged bramble flowers compared to flower buds, as reflected by multiple diversity measures. This suggests that anthesis creates new opportunities for bacterial colonization, through both environmental deposition and insect visitation. Notably, the distinct bacterial beta diversity observed in open and bagged flowers compared to flower buds appeared to result from higher within-group variations in these flower groups, indicating that both insect-visitation and environmental deposition shape individual-specific and heterogeneous bacterial assemblage, and reflect stochastic bacterial colonization processes associated with anthesis. In addition, bacterial load was significantly higher in open flowers compared to bagged flowers and flower buds, suggesting that insects are likely the major source of floral bacteria. This interpretation is consistent with previous studies reporting that pollinator visitation enhances the abundance of culturable bacteria in flowers [82, 83].

Compared to bacterial community, the diversity of floral fungal community was influenced by insect visitation and environmental deposition in a slightly different way. The floral fungi appeared to originate mainly from environmental deposition according to the results from quantitative PCR. This is supported by the high diversity of fungal spores present in the atmosphere and the observation that fungi are more abundant than bacteria in outdoor air, comprising 92.5% of viable airborne organisms [84, 85]. In contrast, insect visitation had a minor effect on fungal load, as indicated by the lack of significant difference between open and bagged flowers. This limited effect may be explained by the substantially lower fungal loads relative to the bacterial loads associated with insects [86–88]. Our results also show that although insect visitation did not further increase the overall fungal load beyond that contributed by environmental deposition, it introduced distinct insect-associated fungal taxa that altered the community structure, as revealed by beta diversity analysis.

Functional annotation of floral microbes provided additional insights into the horizontal microbial transmission dynamics via bramble flowers. Although no significant differences in fungal functional guilds were identified across the flower groups, we found that insect visitation enriched bacteria with fermentation capability and even pathogenic bacteria. Considering the high bacterial load in open flowers, the abundance of these bacteria could be further magnified. Some insects are associated with fermentative bacteria in their digestive tract; thus, these bacteria could be transferred to flowers during insect foraging activity [80, 89, 90]. We also noted that not all bacterial genera could be annotated using the latest version of FAPROTAX database (version 1.2.10 released in 2024). For example, several honeybee-associated bacterial genera were missing such as *Bombella* and *Gilliamella*. These bacteria also possess fermentation capability [91, 92], therefore the fermentation group in our analysis may still be underestimated.

For functional annotation using the FAPROTAX and FungalTraits databases, some major limitations also exist. It should be emphasized that these annotations are predictions based on genus-level taxonomy and literature-documented capabilities, not real measurements of function in our system [57, 58]. Thus, future work incorporating metatranscriptomics, metabolomics, or culture-based functional assays would be needed to validate the predicted functional capabilities. The annotation process always extrapolates traits from cultivated representatives to all members of a genus including uncultured microbes, which can be wrong as more diverse strains are described [93]. For example, some large microbial genera such as *Pseudomonas* (*Gammaproteobacteria*) and *Cladosporium* consist of hundreds of species with diverse lifestyles [94, 95], and limited by taxonomic resolution, the exact species composition and their associated functions within the samples cannot be determined. In addition, many fungi exhibit a continuum of life-history strategies across different trophic modes, and vary depending on environmental conditions [96], which further affect the accuracy of functional predictions. Therefore, the functional annotation results should be viewed skeptically until validated by complementary approaches.

Co-occurrence networks are important tools to explore microbial interactions. In plant-associated microbial networks, hub microbes often play dominant roles in interacting with other microbes, and disturbances to hubs may result in shift in the overall microbiota structure [97]. Our co-occurrence network analysis revealed that the flower bud networks were relatively sparse and isolated compared to the open flower and bagged flower networks, consistent with the patterns observed in multiple diversity measures. Although several microbial hub genera were identified in the flower bud networks, it should be noted that the identification was based on proportion, and they in general showed lower node degrees compared to those in open and bagged flowers. Therefore, the microbial hubs in flower buds might lack the expected levels of inter-taxa connectivity. For example, *Buchnera* (*Gammaproteobacteria*) was designated a bacterial hub in flower buds; however, this genus comprises a single species, *Buchnera aphidicola*, an obligate endosymbiont of aphids with limited capacity for interactions with non-endophytic floral microbes [98]. The networks of bagged and open flowers exhibited distinct topological properties. Bagged flower networks displayed greater connectivity, likely because environmental deposition introduces diverse microbial taxa but with low absolute loads, allowing sufficient nutritional resources to foster inter-taxa interactions. The centralization observed in open flower networks might be attributed to microbial hubs exclusively introduced by pollinator insects and their high microbial load, such as *Neokomagataea* and *Metschnikowia* (they were also the only differentially abundant genera in the open flower networks). This finding is consistent with previous studies indicating that these two microbial genera inhabit nectar environment and are more abundant in insect-visited flowers [83]. It has been shown that *Metschnikowia* co-occurs with *Acinetobacter* spp. and is able to deplete glucose and enrich fructose which is preferentially consumed by the bacteria [99, 100]. It could also increase the pollinator visitation rate of a bumblebee-pollinated vine *Clematis akebioides*, possibly through the production of volatiles [101]. Similarly, *Neokomagataea* exhibits metabolic effects of changing saccharide and amino acid concentrations [102]. Its close relative *Gluconobacter* has also been reported to reduce nectar foraging activity of birds [103, 104]. Collectively, these findings suggest that insect visitation introduces pivotal microbial hubs that reshape floral microbiota interactions, potentially via altering floral chemical profiles and plant-pollinator dynamics.

Our analysis revealed that some hub genera in the single-domain networks were not retained as hubs in the cross-domain network. This suggests that their influence may be limited to intra-domain interactions and not extend to cross-domain dynamics. Conversely, hubs in the cross-domain network may represent genera that bridge bacterial and fungal communities, playing roles in inter-domain ecological processes. *Cladosporium* was a hub fungus in the cross-domain networks but not in the fungal networks, and it was the only hub consistently identified across all flower groups. This fungal genus shows either endophytic or epiphytic lifestyle in plants [105, 106], and is capable of producing a group of plant growth-promoting volatiles [107]. However, its interaction with bacteria in plant-associated niches is still unclear, and we suggest that future studies should investigate the potential cross-domain interactions involving *Cladosporium* to better understand its ecological roles in floral microbiota.

In conclusion, by characterizing and analyzing the microbial profiles of open flowers, bagged flowers and flower buds of a highly insect-visited hub plant bramble, we showed that the microbial diversity and structure are substantially influenced by environmental deposition and insect visitation after anthesis. Our results demonstrate that insect visitation is the dominator influencer of bramble floral microbiota, through altering microbiota structure and functional properties, increasing microbial load, and introducing hub microbes that reshape microbial interactions. Based on these findings, we recommend that hub plants receive more research attention, as they may play key roles in horizontal microbial transmission across different ecosystems. Future studies focusing on functional interactions among floral microbes will also be essential to fully understand the ecological consequences of plant–microbe–pollinator networks.

## Supplementary Information

Additional file1 (XLSX 38 kb)

Additional file2 (PDF 1213 kb)

## Data Availability

The sequencing data are available at the NCBI GenBank database under the BioProject PRJNA1261409 and PRJNA1261414. Additional information is available in the supplementary files.
