# Supplementary material for: Microbiota and horizontal microbial transmission dynamics associated with bramble (Rubus spp.) flowers
Source: Environ Microbiome. 2026 Jan 17;21:31. doi: 10.1186/s40793-026-00853-3 (PMC12895733; doi:10.1186/s40793-026-00853-3)
Supplement: Supplementary file 2 — Additional file2 (PDF 1213 kb) [file 40793_2026_853_MOESM2_ESM.pdf]

Supplementary file 2

Microbiota and horizontal microbial transmission dynamics associated with bramble (*Rubus* spp.) flowers

Haoran Shi[1*], Stefan Ratering[1], Bellinda Schneider[1], Dirk Höper[2] and Sylvia Schnell[1]

[1]Institute of Applied Microbiology, Justus-Liebig University Giessen, IFZ Heinrich-Buff Ring 26, 35392 Giessen, Germany

[2]Friedrich-Loeffler-Institut, Federal Research Institute for Animal Health, Südufer 10, 17493 Greifswald - Insel Riems, Germany

***Corresponding author:** Haoran Shi, Institute of Applied Microbiology, Justus-Liebig University, IFZ, Heinrich-Buff-Ring 26-32, 35392, Giessen, Germany. E-mail: Haoran.Shi@umwelt.uni-giessen.de

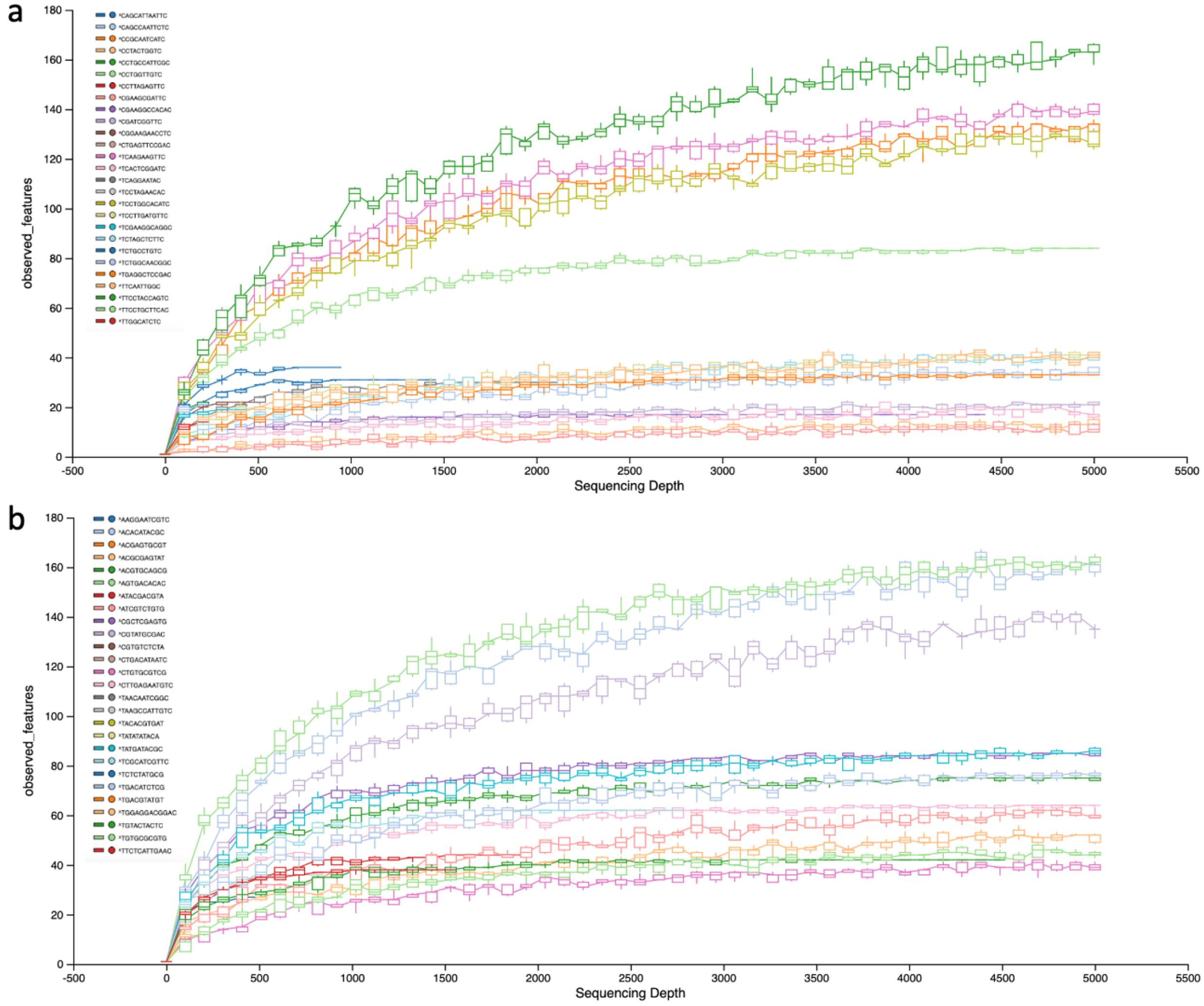

Figure S1. Rarefaction curve illustrating observed features versus sequencing depth for bacterial (a) and fungal (b) datasets. Rarefaction was performed from 0 to 5000 reads in 50 evenly spaced steps with 10 iterations per step.

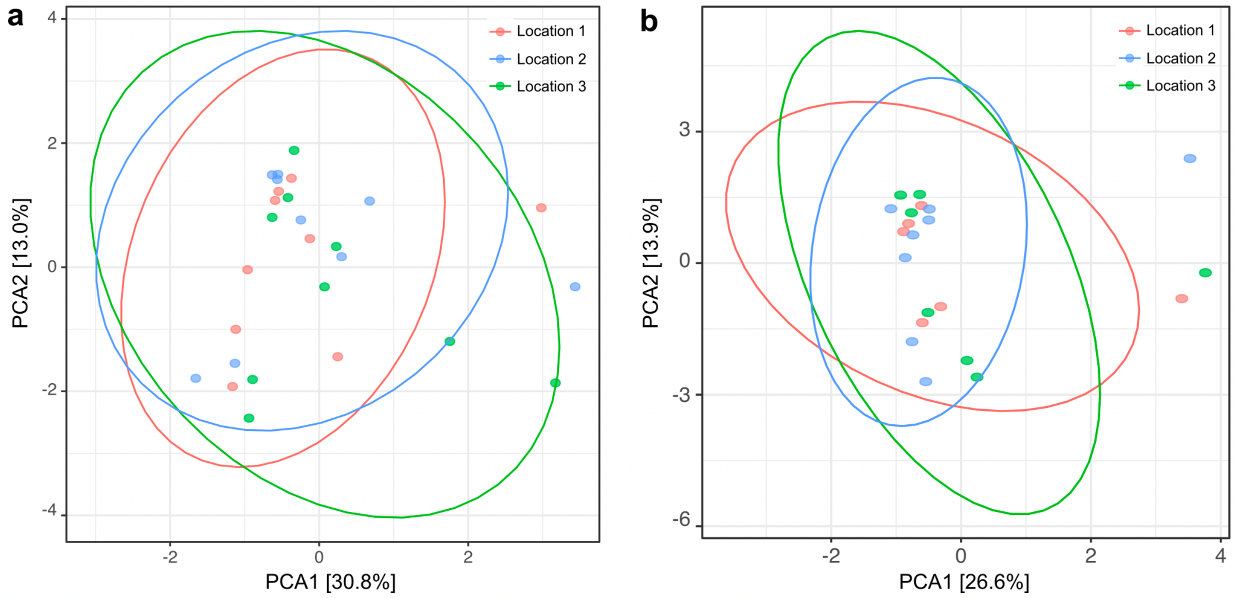

Figure S2. Beta diversity analysis of bacterial (**a**) and fungal (**b**) communities at genus level across different sampling sites based on robust Aitchison distances. The ellipses represent the 95% confidence intervals for each group.
